# Urinary Bladder Diverticulum: A Single-Center Experience in the Management of Refractory Lower Urinary Symptoms Using a Robotic Platform

**DOI:** 10.7759/cureus.42354

**Published:** 2023-07-24

**Authors:** Sarosh Janardanan, Anurag Nigam, Dimitrios Moschonas, Matthew Perry, Krishna Patil

**Affiliations:** 1 Department of Urology, Ashford and St Peter’s National Health Services Foundation Trust, Chertsey, GBR; 2 Department of Urology, Royal Surrey County Hospital, Guildford, GBR

**Keywords:** lower urinary tract symptoms, bladder diverticulum, robotic surgery procedures, post void residual bladder volume, uti: urinary tract infection, bladder dysfunction

## Abstract

Introduction

Urinary bladder diverticulum (UBD) is commonly seen in urological practice and, in most cases, does not need treatment specifically directed towards it. However, it can give rise to symptoms that are not distinct from this finding. This makes the evaluation and management of this complex patient group challenging. We present our experience with robotic bladder diverticulectomy (RBD) for acquired bladder diverticulum to assess the outcomes and safety of this procedure when patient symptoms have failed to respond to either medical or surgical treatment directed at other associated contributing factors.

Methods

We retrospectively collected data on all patients who underwent RBD for persistent lower urinary tract symptoms (LUTS) at Royal Surrey County Hospital, Guildford, between 2016 and 2021, including baseline characteristics, urodynamic findings, intraoperative and postoperative outcomes, and a six-month follow-up. Patients who were diagnosed with cancer in the diverticulum, associated pathology that may contribute to their symptoms, or who had concomitant procedures at the time of RBD were excluded from this study.

Results

We had six patients who underwent RBD; the median age and body mass index (BMI) were 63.8 years (range 48-73) and 27.1 kg/m^2 ^(range 24-32), respectively. The most common presenting symptoms were refractory LUTS and recurrent urinary tract infections (UTIs). The urodynamic evaluation revealed varying findings like bladder outlet obstruction (BOO), poor compliance, and equivocal readings in these patients. All patients reported incomplete bladder emptying and double voiding, with half practicing clean intermittent self-catheterization (CISC). Diverticulum size averaged 9.4 cm (range 8.5-12). The median operative time and blood loss were 166 mins (range 150-180) and 75 mls (range 50-100), respectively. The average length of stay was 1.6 days (range 1-3). Three patients developed UTIs within a month after surgery, requiring a course of oral antibiotics. Post-void residual (PVR) measured an average of 32.6 mls (range 0-161) postoperatively compared to a preoperative average of 249 mls (range 125-400), showing a two-tailed p-value of 0.016. The International Prostate Symptom Score (IPSS) score for these patients showed an average of 27.83 (range 24-31) preoperatively compared to the postoperative average of eight (range 7-12), showing a two-tailed p-value of 0.0001. Final histology showed no malignancy, and all patients reported symptom improvement, with none requiring CISC after surgery.

Conclusion

RBD is a safe and effective procedure in carefully selected patients with refractory LUTS and UTIs showing good postoperative and functional outcomes. The presence of a large diverticulum can have a complex effect on bladder dynamics. In the era of robotic surgery and enhanced recovery, discussion about diverticulectomy should be encouraged after proper evaluation and counseling for patients who have failed to improve with other measures of treatment for their symptoms.

## Introduction

Bladder diverticulum is a common finding noted in everyday urological practice. These are defined as herniations of the bladder urothelium through its muscular wall. Due to the absence of muscle cover, this out-pouching fails to empty spontaneously. This, in turn, leads to stagnation of urine, causing a myriad of symptoms, including troublesome LUTS, double voiding, incomplete emptying, dysfunctional voiding, recurrent infections, bladder calculi, and cancer [1]. They can rarely cause symptoms related to pressure on adjacent structures if they are very large. Bladder diverticulum (BD) could be the primary non-obstructive type (congenital) or secondary (acquired) to BOO or neurogenic bladder [2]. When indicated, the treatment is largely surgical, using an open, laparoscopic, endoscopic, or robotic approach. Most of the available literature on bladder diverticulum is based on points of technique, with no high-quality experimental studies to suggest how these should be managed when associated with symptoms that have failed to improve with other forms of treatment. We have excluded patients with cancer and those who had concomitant procedures performed at the same time, giving an accurate effect of this procedure on patient symptoms. All studies in the literature have included patients either with cancer in the diverticulum or cases where additional procedures like BOO surgery, reimplantation, or prostatectomy were performed, making it difficult to ascertain the effect of RBD on patient symptoms. We report our experience of managing patients with bladder diverticulum suffering from persistent symptoms despite treatment using the robotic, intra-peritoneal, and extra-vesical approach [3].

## Materials and methods

Study design

The data of patients who underwent RBD (intraperitoneal, extravesical approach) for persistent LUTS at Royal Surrey County Hospital, Guildford, over five years between 2016 and 2021 were retrospectively collected and reviewed. 

**Figure 1:**
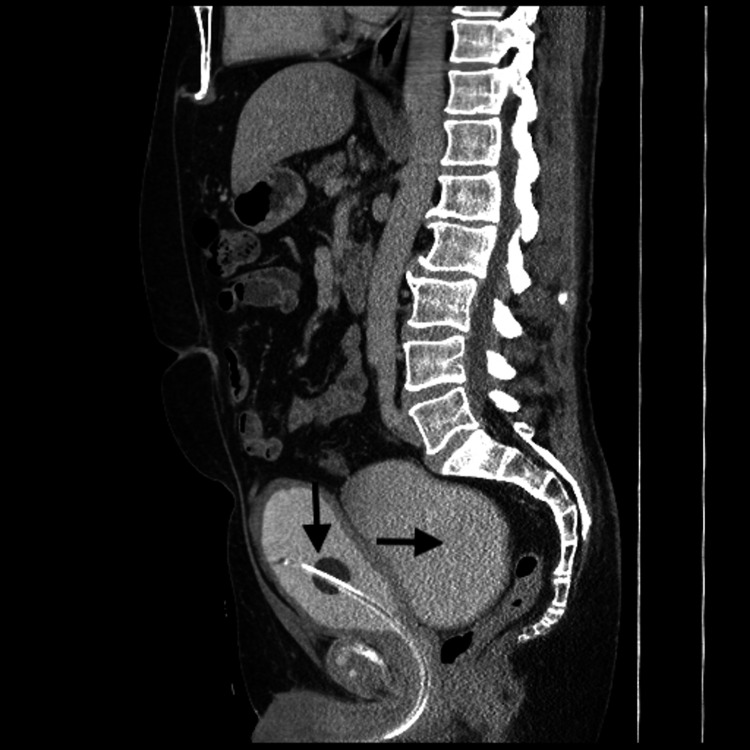
Sagittal view of CT abdomen and pelvis with contrast showing a large posterior bladder diverticulum (horizontal arrow) with a catheter balloon noted in the bladder (vertical arrow)

**Figure 2:**
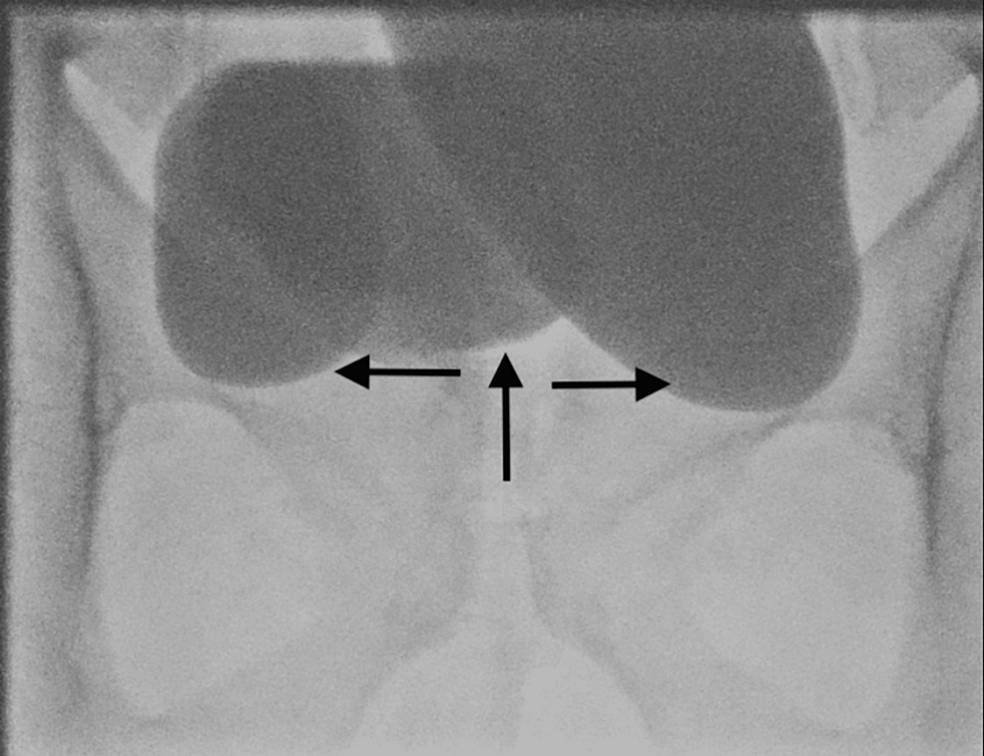
Coronal view at the time of video urodynamics shows bilateral diverticulum (horizontal arrows) with a small bladder capacity (vertical arrow)

After careful history and examination, all patients were evaluated with endoscopy and imaging (Figure 1) to identify the position and size of the diverticulum. The location of the diverticular neck in relation to ureteric orifices and the presence of other associated pathology were noted. All patients underwent urodynamic (Figure 2) evaluation for assessment of the functional status of the bladder during the filling phase and the presence of bladder outlet obstruction during the voiding phase. 

Inclusion and exclusion criteria

Patients who were found to have associated pathology that may contribute to their symptoms, like the presence of malignancy, urethral strictures, benign prostatic hyperplasia, a high bladder neck, or stones, were excluded from the study. Only patients with acquired bladder diverticulum with no other associated contributing pathology were included in this study.

Data collection and statistical analysis

Baseline patient characteristics like age, American Society of Anesthesiologists (ASA) score, BMI, and previous surgeries were included, along with presenting complaints, PVR, urodynamic parameters, and size of the diverticulum. Intraoperative details like operative time (OT), blood loss (BL), and use of a stent or drain were noted. Post-operative features, including length of stay (LOS), timing of stent removal, and details of complications at one-month and three-month follow-ups, were included. The histological diagnosis and postoperative outcomes for a six-month follow-up period were reviewed.

The pre- and postoperative parameters, like the IPSS score and PVR, were compared using the paired t-test to evaluate the p-value and statistical significance of these findings.

## Results

We had a total of six patients who underwent RBD for persistent symptoms despite medical and surgical treatment directed at other possible contributing factors like bladder overactivity or bladder outlet obstruction. The median age and BMI of these patients were 63.8 (range 48-73) and 27.1 (range 24-32), respectively. The median preoperative PVR was noted to be 249 mls (range 100-400). The median size of the diverticulum was 9.4 cm (range 8.5-12). Three patients (50%) had their diverticulum arising from the left lateral wall, two (33.33%) from the right lateral wall, and one (16.66%) had a bilateral diverticulum, with the left noted to be larger than the right. The average preoperative IPSS score was noted to be 27.83 (range 24-31).

Although these patients presented with varying symptoms, the predominant one in three (50%) of them was voiding, with an average maximum flow rate (Q max) of seven (range 3-11). Two (33.33%) patients suffered from recurrent urinary tract infections (UTI), and one (16.66%) presented mainly with refractory storage LUTS. Five (83.33%) out of six patients had surgery for BOO, which failed to improve their symptoms. Of these, three (50%) patients underwent transurethral resection of the prostate (TURP), one (16.66%) patient underwent green light photo vaporization of the prostate (GL-PVP), and bladder neck incision (BNI). None of these BOO procedures were performed in the last six months. Three (50%) patients were performing clean intermittent self-catheterization (CISC) to aid bladder emptying.

On urodynamic (UDS) evaluation of these patients, two (33.33%) patients had findings of bladder outlet obstruction. Four (66.66%) patients showed normal bladder compliance and end detrusor filling pressures. Two (33.33%) patients had poorly compliant bladders, and all (100%) patients failed to empty their bladders. The average preoperative IPSS score was 27.83 (range 24-31).

**Figure 3:**
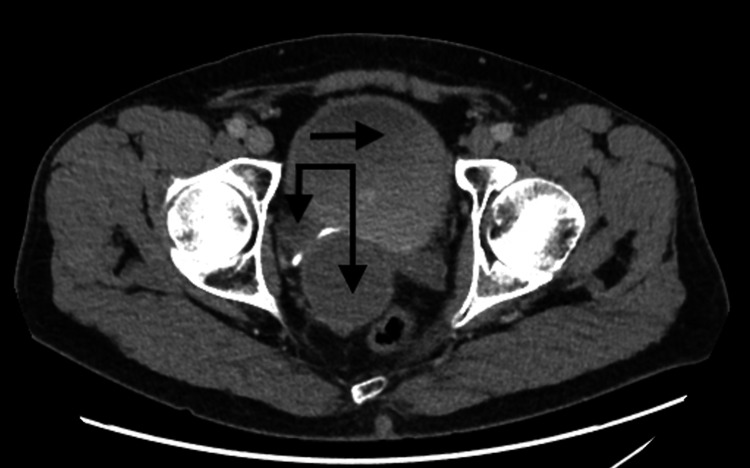
Transverse view of CT abdomen and pelvis with contrast showing bladder diverticulum (vertical arrows) noted wrapped around the distal ureter as it enters the bladder, marked with a horizontal arrow

The ASA score for all patients was two. The median operative time was found to be 166 minutes (range 150-180), and the average estimated blood loss was 75 mls (range 50-100). Five (83.33%) patients had elective ureteric stents placed intraoperatively, which were removed two weeks later using flexible cystoscopy, except in one, where they were left for six weeks as the diverticulum was wrapped around the distal end of the ureter and involved more extensive dissection (Figure 3). All patients had an intraperitoneal drain and a urinary catheter inserted, which were removed on postoperative days 1 and 14, respectively.

The average length of stay (LOS) was calculated as 1.6 days (range 1-3). A post-operative cystogram was performed in two (33.33%) patients before the removal of the catheter, which included the patient with a poorly compliant bladder (patient two) on urodynamic evaluation and the one who had the diverticulum wrapped around the distal end of the ureter (patient four) to confirm complete healing as a measure of caution. 

The average PVR postoperatively was 32.6mls (range 0-161). This difference was found to be statistically significant when compared to the preoperative average of 249 mls using the paired t-test at the 95% confidence interval, showing a two-tailed p-value of 0.0161. The average IPSS score postoperatively was eight (range 7-12). This difference was noted to be statistically significant when compared to the preoperative average of 27.83 using the paired t-test at the 95% confidence interval, showing a two-tailed p-value of 0.0001.

Three patients (50%) developed urinary infections within 30 days of surgery, which were treated with a course of oral antibiotics as outpatients (Clavien-Dindo grade 2 complication). At 90 days of follow-up, none of the patients reported any other complications. On histological analysis, none of the patients were found to have cancer, and the most common finding noted was squamous metaplasia or dysplasia. All patients reported improvement in their overall quality of life and have been symptom-free at the six-month follow-up, with none needing CISC (Table 1).

**Table 1:** Patient characteristics, operative details, and postoperative outcomes. UTI: Urinary tract infection, LUTS: Lower urinary tract symptoms, CISC: Clean intermittent self-catheterization, GL-PVP: Greenlight photo vaporization of prostate, TURP: Transurethral resection of the prostate, BNI: Bladder neck incision, PVR: Post-void residual urine, ASA: American Society of Anesthesiologists, BMI: Body mass index, IPSS: International prostate symptom score, UDS: Urodynamics, LOS: Length of stay

	Patient 1	Patient 2	Patient 3	Patient 4	Patient 5	Patient 6
Age (years)	73	62	68	66	66	48
Presenting symptom	UTIs	LUTS+CISC	UTIs	LUTS+CISC	LUTS+CISC	UTI+LUTS
Previous surgery	GL-PVP	BNI	TURP	TURP	TURP	-
PVR (mls) before surgery	255	394	100	400	125	220
ASA score	2	2	2	2	2	2
BMI (kg/m2)	25	27	26	32	24	29
Size (cm)	8.5	12	6	12	12	6
Location	Left	Right	Left	Bilateral	Right	Right
IPSS score before surgery	31	30	30	27	25	24
UDS filling phase	Normal	Reduced compliance	Normal	Reduced compliance	Normal	Normal
UDS voiding phase	Obstructive	Obstructive	Equivocal	Equivocal	Equivocal	Equivocal
Operative time (mins)	175	150	175	180	150	166
Blood loss (mls)	100	75	50	75	75	75
LOS (days)	2	1	3	1	1	2
PVR after surgery (mls)	0	0	15	20	0	161
IPSS score after surgery	7	8	7	7	7	12
Postoperative complication	UTI	-	-	-	UTI	UTI
Histology	Follicular cystitis and squamous metaplasia	Inflammatory fibrosis	Squamous dysplasia	Squamous dysplasia	Squamous metaplasia	Squamous metaplasia

## Discussion

Definition and pathophysiology

The bladder diverticulum is described as an out-pouching of bladder urothelium through its muscular layer to form a redundant pocket not capable of emptying its contents due to a lack of muscle covering. Although this rarely causes concern, it may occasionally lead to refractory LUTS, recurrent UTIs, bladder calculus, and neoplastic change. These occur due to a combination of abnormal micturition function, detrusor coordination, and contraction [4]. The chances of developing neoplastic changes in a bladder diverticulum are reported to be between 1% and 10% [5]. The above-mentioned features form an indication for surgical treatment of this otherwise indolent finding. As they most commonly develop secondary to BOO, a vast majority of these patients respond well to measures directed to relieve the obstruction. We only selected patients who failed to improve with all other measures for robotic bladder diverticulectomy.

Careful evaluation of these patients with endoscopy, imaging, and urodynamic studies is needed for proper counseling before surgery. Increased pressures due to low compliance can give rise to the formation of a diverticulum, and a diverticulum itself can lead to reduced compliance, causing a cycle of worsening LUTS. Urodynamic studies help in determining the presence of detrusor overactivity, detrusor failure, or bladder outlet obstruction. An experimental animal study on rabbits with bladder diverticulum demonstrated alteration of bladder filling pressures leading to detrusor overactivity. It also showed increased bladder wall connective tissue, leading to reduced bladder compliance and increased wall thickness. Most patients with secondary diverticulum tend to have high voiding pressures as opposed to primary bladder diverticula. These patients also tend to have high PVR due to the ease of emptying into the diverticulum neck instead of the bladder neck [5]. As was seen in our cohort, patients with poor bladder compliance also show good improvement in their symptoms, as the effect of the diverticulum on bladder dynamics is complex. The information gained from a urodynamic evaluation is important for counseling the patient on possible outcomes after surgery. A recently performed animal study reported a further reduction in compliance in addition to the effects of pre-existing BOO due to the presence of bladder diverticulum [6]. As most patients show good improvement with measures tailored to any contributing factors like BOO, this should be addressed first. Our patient cohort had persistent symptoms with no other cause attributed to them. Urodynamic findings should be carefully discussed with the patients, along with possible postoperative outcomes, before offering surgery.

Operative approaches

Various techniques have been described for surgically treating this condition, including trans-abdominal extra-peritoneal or intra-peritoneal repair, which can be performed using an open, laparoscopic, or robotic approach. It can also be managed using an endoscopic approach with fulguration or resection techniques in patients who are unfit to have a more extensive procedure [7]. It is known that a minimal access approach is better than an open approach when comparing parameters like operative time, blood loss, the need for post-operative analgesics, and length of hospital stay [8]. Apart from the known benefits of minimal access surgery, the robotic approach offers an additional advantage in being able to access difficult areas with greater magnification and depth perception, allowing instrumentation with seven degrees of freedom.

Robotic bladder diverticulectomy was first reported by Berger and Stifelman in 2006 [9]. Various modifications of this approach have been described, which mainly involve the technique used in identifying the diverticulum. Myer et al. described the technique of performing a cystoscopy and ureteric stenting before the procedure to aid early identification of the ureter and safeguard it [10]. The bladder diverticulum can also be identified by the illumination of the cystoscope or the use of a catheter with a balloon inflated in the diverticulum with a narrow neck [11]. The use of methylene blue dye and firefly technology has also been used for this purpose [12]. We have not described the operative approach of the intraperitoneal extravesical robotic approach, as this has been elaborated upon as a point of technique by Ashton et al., 2019 [3].

Literature review

On reviewing the literature, nine studies [7,10,12-18] with more than five patient series were included for comparison of outcomes. Most of the parameters in the current cohort were either comparable or better than those reported in similar studies. However, we found an increased incidence of postoperative UTI in our group compared to other series. There were no reversible risk factors found to account for these. None, except one study, reported Clavien-Dindo grade 3 or 4 complications [17] in the postoperative period, supporting the safety of this procedure. All studies have reported good patient satisfaction and outcomes, as was seen in our group, where none of the patients needed CISC or further treatment after surgery. To the best of our knowledge, our series is the first in which RBD was performed for a purely benign acquired bladder diverticulum without the inclusion of additional procedures aimed at BOO in the same sitting. This asserts that the improvement in our patient cohort was unlikely to be attributed to any other compounding factors. All other studies have included patients who underwent other procedures in the same sitting, making it difficult to attribute the benefits to RBD alone since we know that most of these patients do well when their BOO is addressed (Table 2).

**Table 2:** Outcomes from comparable studies in literature RASP: Robot-assisted simple prostatectomy, RARP: Robot-assisted radical prostatectomy, UTI: Urinary tract infection, PLND: Pelvic lymph node dissection, TURP: Transurethral resection of the prostate, TUIP: Transurethral incision of prostate, PVP: Photo vaporization of the prostate.

Study	Patients	Average Size of Diverticulum	Operative approach/ additional procedures/ Cancer	Operative Time (min)	Blood Loss (ml)	Length of Stay (days)	Complication – 90days
Present series	6	9.4	Extravesical/None/No	166	75	1.6	3 UTI
Develtere et al., 2022 [18]	23	7	Mixed/12 RASP, 1 RARP/Yes	140	250	3	1 UTI, 1 urinary leak
Liu et al., 2021 [17]	20	7.6	Mixed/5 RARP, 4 RASP, 2 PLND, 1 reimplant, 1 enterotomy repair/Yes	184	100	2	1 clot retention, 1 enterotomy during adhesiolysis
Cacciamani et al., 2018 [13]	6	7.1	Extravesical/None/Yes	112.5	25.8	7	1 urinary retention
Tufek et al., 2016 [7]	9	7.2	Not mentioned/9 TURP or PVP/No	186	71	5	None
Davidiuk et al., 2015 [14]	16	7.2	Mixed/9 TURP, 2 TUIP/Yes	241	50	2	None
Abreu et al., 2014 [15]	10		Mixed/1 RARP, 1 RASP, 1 TURP/Yes	210	75	-	None
Moore et al., 2012 [12]	5	2.5-11	Not mentioned/None/Yes	216	45	1	None
Alturende et al., 2011 [16]	6	2.1-7.6	Mixed/1 PLND, 1 ureteric reimplant, 1 TURP/Yes	232	100	3	1 UTI, 2 urinary retentions
Myer et al., 2007 [10]	5	10.8	Extravesical/1 ureteric reimplant, 2 TURP/Yes	178	-	3	None

Limitations 

One of the main limitations of this study is the rather small number of patients, which makes it difficult to validate our results. This is because most patients with bladder diverticulum are asymptomatic, and the ones who become symptomatic rarely need anything more than treatment measures for BOO. A multicenter collaborative study is suggested to obtain larger numbers and have definitive results related to this cohort of patients.

## Conclusions

Robot-assisted bladder diverticulectomy is a safe procedure with good outcomes in a select patient group who continue to suffer from symptoms after having definitive surgery for infra-vesical obstruction. These symptoms rarely respond to any other modality of treatment due to the persistence of the primary problem. This is primarily due to the complex effects that a diverticulum can have on bladder functionality. Proper preoperative counseling based on the urodynamic findings of patients is of utmost importance. Since this group of patients tends to have good outcomes postoperatively, they should be offered definitive surgery when other causes for their symptoms have been ruled out.
